# Blending sludge alkaline hydrolysate and urea affects grape yield and quality by regulating soil bacterial communities

**DOI:** 10.3389/fpls.2025.1665661

**Published:** 2025-09-30

**Authors:** Donghe Xue, Yan Yang, Huofeng Zhang, Yijie Quan, Zejin Li, Zixu Li, Wei Wang, Huijuan Bo, Dongsheng Jin, Minggang Xu, Qiang Zhang, Zhiping Yang

**Affiliations:** ^1^ College of Resources and Environment, Shanxi Agricultural University, Taiyuan, China; ^2^ Soil Health Laboratory in Shanxi Province, Taiyuan, China; ^3^ College of Forestry, Beijing Forestry University, Beijing, China

**Keywords:** bacterial–fungal interactions, sludge alkaline hydrolysate, grape nutrition, sustainable fertilization, yield

## Abstract

**Introduction:**

Fertilization is vital for improving grape (*Vitis vinifera L.*) yield and quality. Unlike traditional nitrogen fertilizers, the mechanisms by which sludge alkaline hydrolysate (SAH), a novel fertilizer, influences grape quality and yield are still poorly understood.

**Methods:**

In this study, six treatments were established: 20% SAH + 80% urea (M1), 40% SAH + 60% urea (M2), 60% SAH + 40% urea (M3), 80% SAH + 20% urea (M4), pure SAH (M5), and pure urea (M6). The effects of applying SAH and urea mixtures to grapes were evaluated, with focus on performance parameters, soil nutrients, and microbial communities.

**Results and discussion:**

The results show that 60–80% SAH application significantly enhanced grape stem thickness, chlorophyll content, photosynthetic efficiency, fruit quality, and increased yield. Concurrently, it elevated soil nutrient contents, improved microbial community structure, and altered nitrogen cycle gene copy numbers. Molecular ecological network analyses indicated that Firmicutes, Acidobacteriota, Gemmatimonadota, and Ascomycota were key taxa. Bacterial–fungal cooperation was the dominant interaction, accounting for 65.98–94.61% of all observed microbial interactions, compared to antagonistic interactions. Mantel analysis showed that bacterial community and nitrogen cycle genes (ammonia-oxidizing bacteria (*AOB*), nitrogen fixation hydrogenase (*nifH*)) were important for grape yield and quality. These findings offer guidance for the effective use of SAH in grape production. Future studies should elucidate how SAH regulates fruit quality-related gene expression to uncover its mechanisms and enable its full-scale use in viticulture.

## 1 Introduction

Grapes (*Vitis vinifera L.*) are a key component of Chinese agriculture. Grape quality and yield are critical for strengthening China’s position in international agriculture. With the rapid development of the grape industry, soil fertility has become a pivotal factor in constraining its sustainable development (Fan et al., 2022). Nitrogen is a key element for the growth and development of grapes, and has significant impacts on plant growth, fruit quality, and soil fertility (Fu et al., 2024). The nitrogen cycle refers to the conversion of nitrogen among various forms. This cycle is regulated by soil microorganisms through the secretion of enzymes encoded by functional genes (Das et al., 2025). These microbial activities influence both soil nitrogen content and plant growth (Han et al., 2024; Zhang et al., 2024a).

In grape cultivation, the application of nitrogen fertilizer plays a crucial role in regulating soil nitrogen content and maintaining the nitrogen cycle (Li and Li, 2025). Nevertheless, the current use of nitrogen fertilizer is accompanied by several challenges, such as the decline in fruit quality caused by over-fertilization and the yield limitations that arise from under-fertilization (Anas et al., 2020; Luo et al., 2024). Traditional grape cultivation often relies on quick-release chemical fertilizers (Kaya et al., 2024). However, the long-term use of these fertilizers can lead to soil acidification and reduced microbial activity (Pahalvi et al., 2021; Gao et al., 2023). Studies have shown that long-term chemical fertilizer use can lower soil pH, inhibit microbial activity, reduce the expression of genes related to the nitrogen cycle (*AOB, nirK*), and affect the sugar-acid ratio and uniformity of fruit coloring (Bai et al., 2023; Kiya et al., 2024). Thus, finding a sustainable and efficient nitrogen fertilizer alternative has become an important research direction in grape cultivation.

Sludge alkaline hydrolysate (SAH), a novel organic fertilizer, is increasingly recognized as a potential alternative to nitrogen fertilizers. It is rich in nitrogen, peptides, proteins, and small-molecule nutrients (Tang et al., 2022). These components help improve soil conditions and enhance fruit quality and yield (Wang et al., 2023). Studies have shown that applying SAH can increase vegetable yields (Wu et al., 2023a). When combined with nitrogen fertilizers, it can significantly boost levels of soluble sugars, organic acids, and vitamin C (VC) in tomato fruits, thereby improving their nutritional value and safety (Xue et al., 2023). Unlike annual vegetables, perennial fruit crops like grapes have more complex soil-microbe-root interactions, making it essential to evaluate long-term SAH impacts in such systems.

Given these gaps, we designed a study to assess the impact of varying SAH and urea ratios on grape performance, soil chemistry, and microbial dynamics. Specifically, the objectives are to (1) evaluate the effects of different treatments on grape performance (stem thickness, leaf area, fruit shape index, yield, chlorophyll, fruit quality and photosynthetic characteristics), (2) elucidate response mechanisms of soil nutrients, microbial communities, and nitrogen-cycling functional genes to different treatments; and (3) identify the key factors influencing grape performance across different treatments. This study demonstrates that blending the novel sludge alkaline hydrolysate fertilizer and urea enhances grape performance through multiple mechanisms, offering a sustainable approach for grape productivity improvement.

## 2 Materials and methods

### 2.1 Overview of the experimental area

This long-term experiment was performed in the TaiGu Comprehensive Experiment Station of the National Grape Industry Technology System in Jinzhong City, Shanxi Province, China (37°25′38.2″N, 112°32′40.5″E). This region features a temperate continental monsoon climate, with an average annual temperature of 10.4°C, annual precipitation of 397.1 mm, a sunshine duration of 2527.5 h, and an altitude of 1098 m. The experiment used a 14-year-old table grape (Zao Hei Bao). The grape plants were planted with a row spacing of 1.30 × 2.12 m and supported on a T-shaped frame.

The SAH was manufactured by Shanxi Jinlian Environmental Science and Technology Co., Ltd. through an alkaline hydrolysis process. By mixing quicklime with domestic sludge, organic nitrogen compounds were extracted to obtain a hot alkaline solution rich in nutrients, including proteins, nitrogen, phosphorus, and potassium. The primary components of this solution are shown in **Supplementary Table S1**. The heavy metal content is significantly lower than the limits stipulated in the Limits for Toxic and Harmful Substances in Fertilizers of the People’s Republic of China (GB 38400-2019).

### 2.2 Experiment design

The present study adopted a randomized experimental design with six treatments based on the principle of equal nitrogen application (176.85 kg/ha), with the application ratios of SAH and urea as follows: 20% SAH + 80% urea (M1), 40% SAH + 60% urea (M2), 60% SAH + 40% urea (M3), 80% SAH + 20% urea (M4), pure SAH (M5), and pure urea (M6). There were six replicates per treatment, resulting in a total of 36 plots, each with an area of 24.80 m^2^ and containing ten grapevines (**Supplementary Figure S1**). The nitrogen contents in urea and SAH were 46.40% and 7.33%, respectively. Following the application rates specified in **Supplementary Table S2**, fertilizer was applied during the stable fruiting (late May) and fruit-swelling (mid-July) periods.

### 2.3 Sample collection and measurement

Five grapevines with similar growth vigor were randomly selected as observation plants within each plot. In June 2024, during the flowering stage of grapevines, three healthy and fully expanded functional leaves were sampled from the upper, middle, and lower strata of every observed plant. Measurements were conducted on the sampled leaves for chlorophyll content, photosynthetic parameters (Ci: intercellular CO_2_ concentration, Tr: transpiration ratio, gs: stomatal conductance, Pn: net photosynthetic rate), as well as leaf length, leaf width and leaf area (LA). Chlorophyll was measured using a handheld SPAD-502 Plus chlorophyll meter (SPAD-502, Konica Minolta, Japan). The photosynthetic indicators were calculated using a LI-6800 photosynthesis instrument (LI-6800, Li-COR, USA). The stem thickness was measured with vernier calipers, and leaf length and width were measured using a tape measure (Li et al., 2022a). The LA was defined as described by Montgomery and Montgomery (1911) Equation 1:


(1) LA = Length × Width × 0.75

where Length is the leaf length, Width is the leaf maximum width, and 0.75 is the LA coefficient.

In September 2024, during grape harvesting, one cluster of fruit was randomly collected from the upper, middle, and lower sections of each observed plant. From each cluster, ten berries were randomly selected, and their fruit length (FL) and fruit diameter (FD) were measured using a vernier caliper. The fruit shape index (FSI) was calculated as the ratio of FL to FD. Subsequently, all berries from each cluster were combined and juiced for fruit quality analysis (Ts: total sugar, Rs: reducing sugars, proteins, VC: Vitamin C) (Bao, 2000). Then, fruits from all six treatments were collected to determine the yield (Y). The following Equation 2 was used:


(2)  Y = m/s×1000 

where m is the total weight of grapes harvested from each treatment, s is the area of each treatment, and 1000 is the conversion factor for m^2^ per hectare.

After the grapes were harvested, we collected soil samples from the 0–20 cm depth in each plot using a five-point sampling method, and then we composited the five samples into one. Soil samples were divided into three sections for real-time fluorescence quantitative PCR (qPCR), high-throughput sequencing after storage at -80 °C, and soil nutrient determination after being air-dried. The method of Bao (2000) was used to determine soil nutrient content (SOC: soil organic carbon, TN: total nitrogen, TP: total phosphorus, TK: total potassium, AP: available phosphorus, AK: available potassium, NH_4_^+^-N: ammonium nitrogen, NO_3_^–^N: nitrate nitrogen).

For soil nutrient indicators, we calculated the membership values using the simple linear scoring method. Adhering to the principle of higher is better, the highest measured value was assigned a membership value of 1. The membership values for other measurements were calculated as their ratios to the highest value (Li et al., 2020; Liebig et al., 2001), using Equation 3:


(3) $$\text{ f }(\text{x})\;=\text{ x}/{\text{x}}_{\text{max}}$$

where f (x) is the membership value, x is the measured value of the indicator, and x_max_ is the highest measured value of the indicator.

### 2.4 DNA extraction, quantitative polymerase chain reaction, and sequence analysis

Soil genomic DNA was isolated with the OMEGA Soil DNA Kit (Omega BioTek, Norcross, GA, USA). Real-time fluorescent qPCR was performed to determine copy numbers of *nifH*, *nirS*, and *AOB* genes using the primers listed in **Supplementary Table S3**. The thermal cycling protocol consisted of initial denaturation at 95°C for 5 min, followed by 40 cycles of 95°C for 15 s and 60°C for 30 s.

The concentration of DNA was quantified using a NanoDrop NC2000 spectrophotometer (Thermo Fisher Scientific, Waltham, MA, USA). Bacterial communities were targeted by amplifying the V3–V4 hypervariable regions with primers 338F (5’-ACTCCTACGGGAGGCAGCA-3’) and 806R (5’-GGACTACHVGGGTWTCTAAT-3’), while fungal communities were assessed through ITS1 region amplification using primers ITS1F (5’-CTTGGTCATTTAGAGGAAGTAA-3’) and ITS2R (5’-GCTGCGTTCTTCATCGATGC-3’). Amplicons were pooled, size-selected via 2% agarose gel electrophoresis, and subsequently purified before quality assessment and quantification. Library preparation was conducted using the TruSeq Nano DNA LT Library Prep Kit (Illumina Inc., San Diego, CA, USA), followed by sequencing on an Illumina NovaSeq 6000 platform (Illumina) with SP Reagent Kit (500 cycles; Illumina). Raw sequences underwent quality control (QIIME2, 2022.11), including filtering, denoising, chimera removal, and assembly. High-quality sequences were clustered into amplicon sequence variants (ASVs) at 100% similarity. Taxonomic assignment of bacterial and fungal ASVs was conducted using the Greengenes2 (https://greengenes2.ucsd.edu/) and UNITE (https://unite.ut.ee/) databases, respectively.

### 2.5 Statistical analyses

One-way Analysis of Variance (ANOVA), followed by Tukey’s HSD test, was performed using IBM SPSS Statistics 21 (IBM Corp., Armonk, NY, USA) software to examine significant differences in grape and soil indicators under different treatments. The ‘linkET’ package (devtoolsinstall_github (“Hy4m/linkET”, force=TRUE) in R (4.4.2; R Foundation for Statistical Computing, Vienna, Austria) was used for Mantel test analysis to explore the relationships between bacterial and fungal communities, nitrogen cycling genes, soil nutrient contents, grape yield, and fruit quality.

To ensure comparability between samples and to reduce the effects of variability in sequencing depth, the microbial data were normalized to a uniform minimum sequencing depth using rarefaction before conducting ecological and network analyses Based on the random matrix theory, network construction was performed for the ASVs of bacteria and ASVs of fungi, and 0.94 was selected as the threshold for each group of bacterial and fungal networks to ensure comparability between the six processed networks (Zhou et al., 2023). We constructed the microbial networks and determined the network parameters using the Molecular Ecological Network Analysis Pipeline (MENAP) (Zheng et al., 2021). The network visualization and generation of node and edge files were performed using the Gephi (v.0.9.2) interactive platform (http://gephi.github.io/). The ZiPi analysis on nodes and edge files was performed using the “ggClusterNet” package in R 4.4.2 (Ding et al., 2016). Based on topological roles defined by within-module connectivity (*Zi*) and among-module connectivity (*Pi*) thresholds, nodes were classified into four categories: (i) peripherals (*Zi* < 2.5, *Pi* < 0.62); (ii) connectors (*Zi* < 2.5, *Pi* ≥ 0.62); (iii) module hubs (*Zi* ≥ 2.5, *Pi* < 0.62); and (iv) network hubs (*Zi* ≥ 2.5, *Pi* ≥ 0.62). Notably, module hubs, network hubs, and connectors were identified as keystone species in the microbial network (Wen et al., 2022).

## 3 Results

### 3.1 Grape performance under different treatments

**Figure 1:**
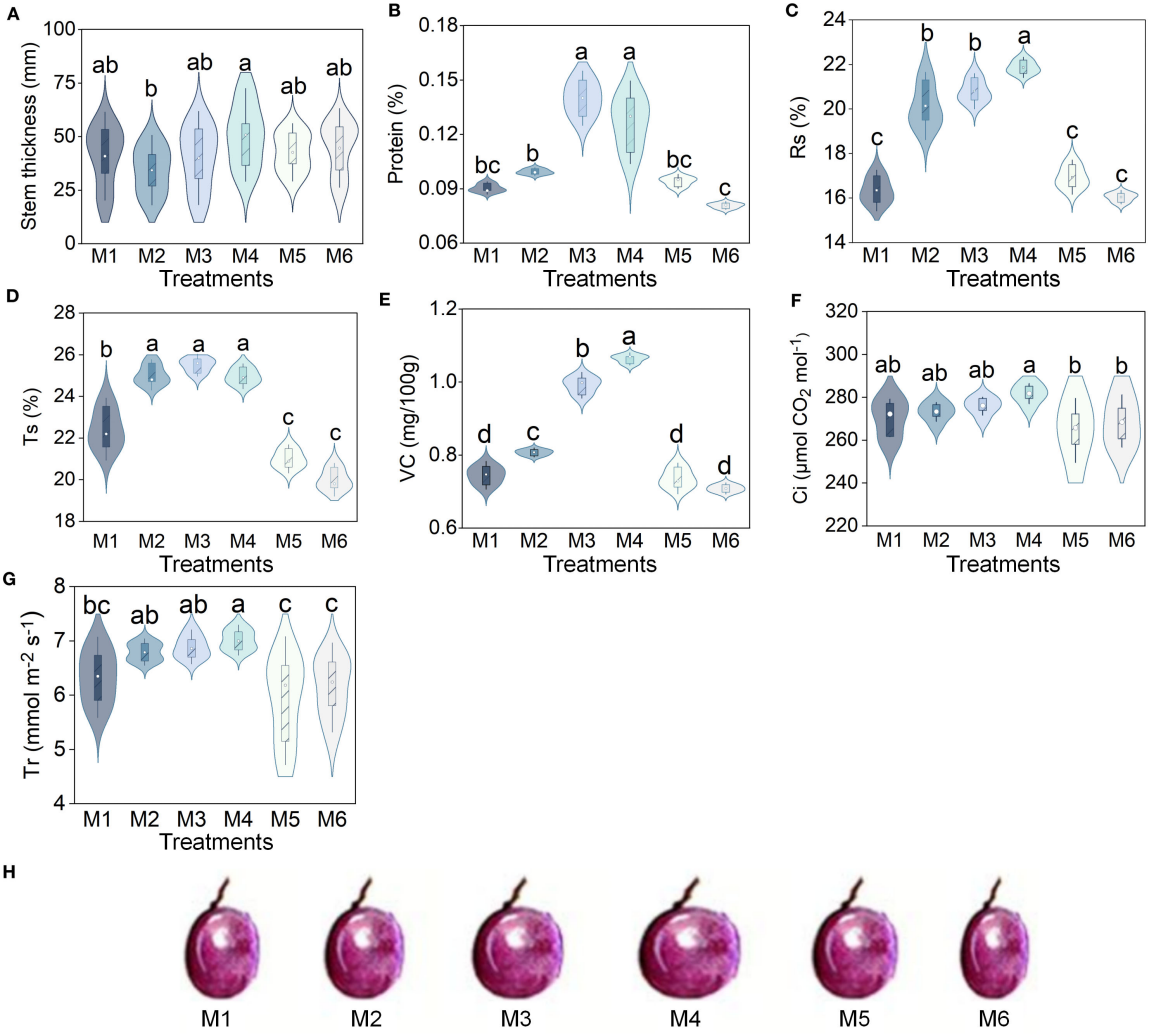
Grape performance under different treatments. **(A)** Stem thickness **(B)** Protein. **(C)** Rs, reducing sugars. **(D)** Ts, total sugars. **(E)** VC, vitamin C. **(F)** Ci, intercellular CO_2_ concentration. **(G)** Tr, transpiration ratio. **(H)** Schematic diagram of fruit type. The treatments included 20% SAH + 80% urea (M1), 40% SAH + 60% urea (M2), 60% SAH + 40% urea (M3), 80% SAH + 20% urea (M4), pure SAH (M5), and pure urea (M6). Different letters indicate significant differences among treatments (*P* < 0.05, n = 6, Tukey HSD). SAH, sludge alkaline hydrolysate.

The M4 treatment achieved maximal values for stem thickness (50.78 cm), leaf area (LA, 98.81 cm^2^), fruit shape index (FSI, 1.05), and yield (48,107.27 kg/ha), with the yield being 99.80% greater than that in M6 (*P* < 0.05, n=6; **Figure 1A**; **Table 1**). In addition, there was no significant difference in chlorophyll among treatments (*P* > 0.05, n=6; **Supplementary Figure S2**).

**Table 1:** Grape performance under different treatments.

Treatment	Leaf length	Leaf width	LA	FL	FD	FSI	Shape	Y
	(cm)	(cm)	(cm^2^)	(cm)	(cm)			(kg/ha)
M1	9.00 ±	9.70 ±	67.58 ±	25.69 ±	22.23 ±	1.15 ±	Oblate	30432.10 ±
	3.08d	1.57e	7.61e	0.80a	0.64bc	0.01b		2178.79b
M2	8.62 ±	10.61 ±	61.72 ±	25.69 ±	22.59 ±	1.13 ±	Oblate	43950.87 ±
	0.48e	1.39c	6.23f	1.16a	0.99 b	0.01b		945.05a
M3	11.01 ±	12.23 ±	88.34 ±	25.55 ±	23.58 ±	1.08 ±	Oblate	45551.38 ±
	1.76a	1.13a	5.04c	1.12a	1.00a	0.01c		6210.93a
M4	11.23 ±	10.08 ±	98.81 ±	24.1 ±	22.85 ±	1.05 ±	Orbicular	48107.27 ±
	1.72a	0.52d	4.18a	1.06b	0.99ab	0.01d		2432.41a
M5	9.42 ±	11.65 ±	71.00 ±	25.62 ±	22.77 ±	1.12 ±	Oblate	26323.57 ±
	1.01c	0.78b	6.16d	0.74a	0.90ab	0.03b		2440.78bc
M6	9.89 ±	9.58 ±	91.13 ±	25.29 ±	21.14 ±	1.19 ±	Oblate	24077.91 ±
	1.22b	0.73f	3.15b	1.01ab	1.05c	0.03a		2244.56c

The treatments included 20% SAH + 80% urea (M1), 40% SAH + 60% urea (M2), 60% SAH + 40% urea (M3), 80% SAH + 20% urea (M4), pure SAH (M5), and pure urea (M6). Different letters indicate significant differences among treatments (*P* < 0.05, n = 6, Tukey HSD). LA, leaf area; FL, fruit length; FD, fruit diameter; FSI, fruit shape index; Y, yield.

Fruit quality (protein, Rs: reducing sugars, Ts: total sugar, VC: Vitamin C) showed a unimodal trend across treatments. The M4 treatment yielded the highest levels of Rs and VC (21.87% and 1.06%), which were 36.43% and 49.30% greater than those in the M6 treatment. Meanwhile, the M3 treatment produced the highest protein and Ts contents (0.14% and 25.50%), exceeding the M6 values by 48.94% and 27.50%. (*P* < 0.05, n=6; **Figures 1B–E**).

Photosynthetic characteristics (Tr, Ci) increased with rising SAH ratios up to M4, after which they declined. The Tr (6.97 mmol m^−2^ s^−1^) and Ci (281.75 µmol CO_2_ mol^−1^) concentrations were both highest under the M4 treatment, which was significantly increased by 12.60% and 17.94% over the values recorded in M5 and M6, respectively (*P* < 0.05, n=6; **Figure 1**). The gs and Pn content did not differ significantly among treatments (*P* > 0.05; **Supplementary Figure S3**).

### 3.2 Characterization of soil nutrients under different treatments

**Figure 2:**
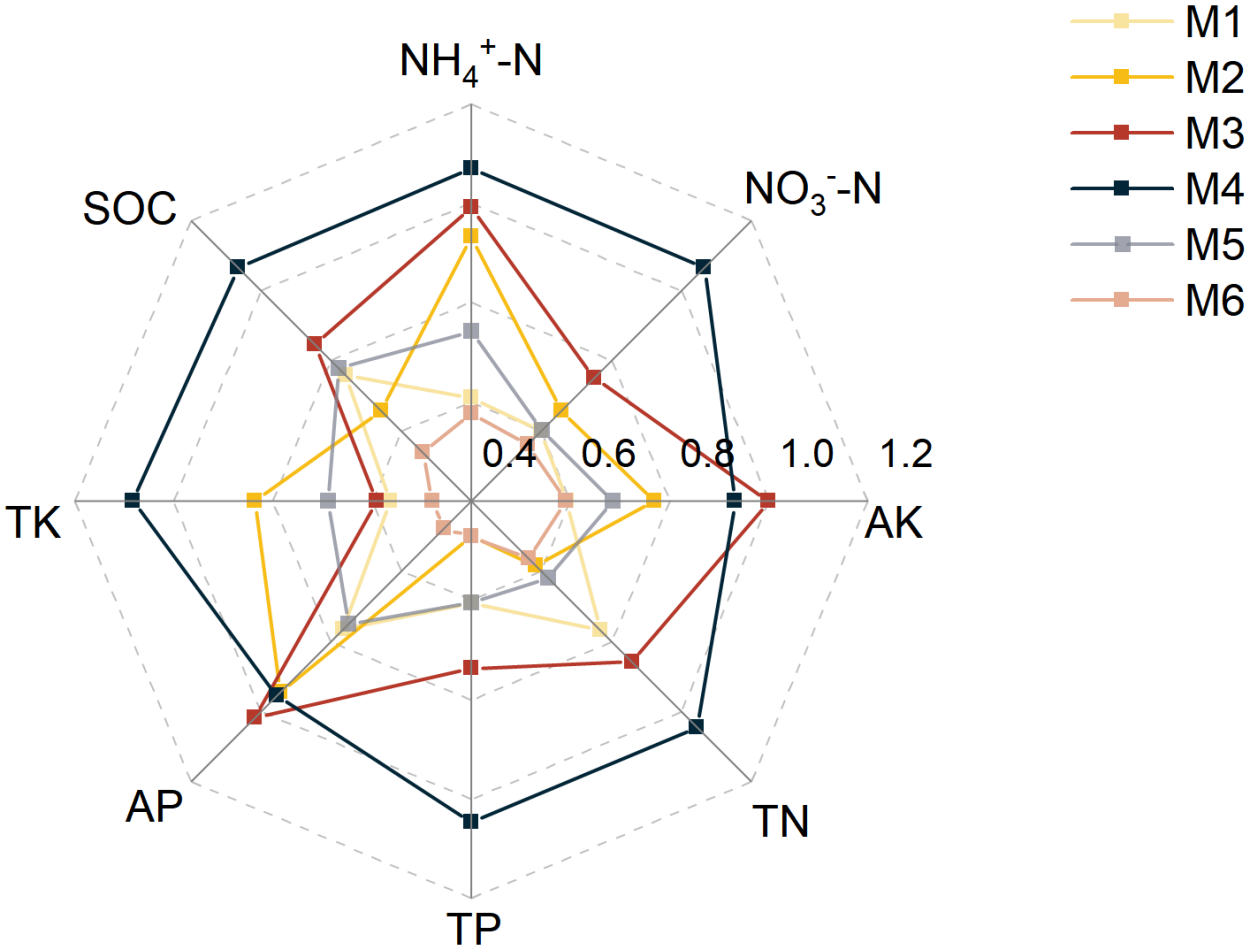
Radar plot of membership value of indicators. SOC, soil organic carbon, TN, total nitrogen; TP, total phosphorus; AP, available phosphorus; TK, total potassium; AK, available potassium; NH_4_^+^-N, ammonium nitrogen; NO_3_^–^N, nitrate nitrogen. The treatment codes, M1-M6, are consistent with those in **Figure 1**.

The contents of SOC (10.03–12.42 g/kg), TN (1.03–1.29 g/kg), TK (19.89–22.62 g/kg), NH_4_^+^-N (3.69–5.92 mg/kg), and NO_3_^–^N (29.10–74.67 mg/kg) were the highest under the M4 treatment and the lowest under the M6 treatment, which increased significantly by 23.83%, 25.24%, 13.73%, 60.43%, and 156.60%, respectively, compared to M6. In contrast, M3 exhibited superior AP (52.78–67.43 mg/kg) and AK (427.71–723.55 mg/kg) levels, exceeding M6 by 27.76% and 69.17% (*P* < 0.05, n=6; **Supplementary Table S4**). Subsequently, the membership degree values of each indicator were calculated, and the results consistently indicated that the soil nutrient conditions were superior under the M3 and M4 treatments (**Figure 2**).

### 3.3 Changes in microbiological characteristics under different treatments

**Figure 3:**
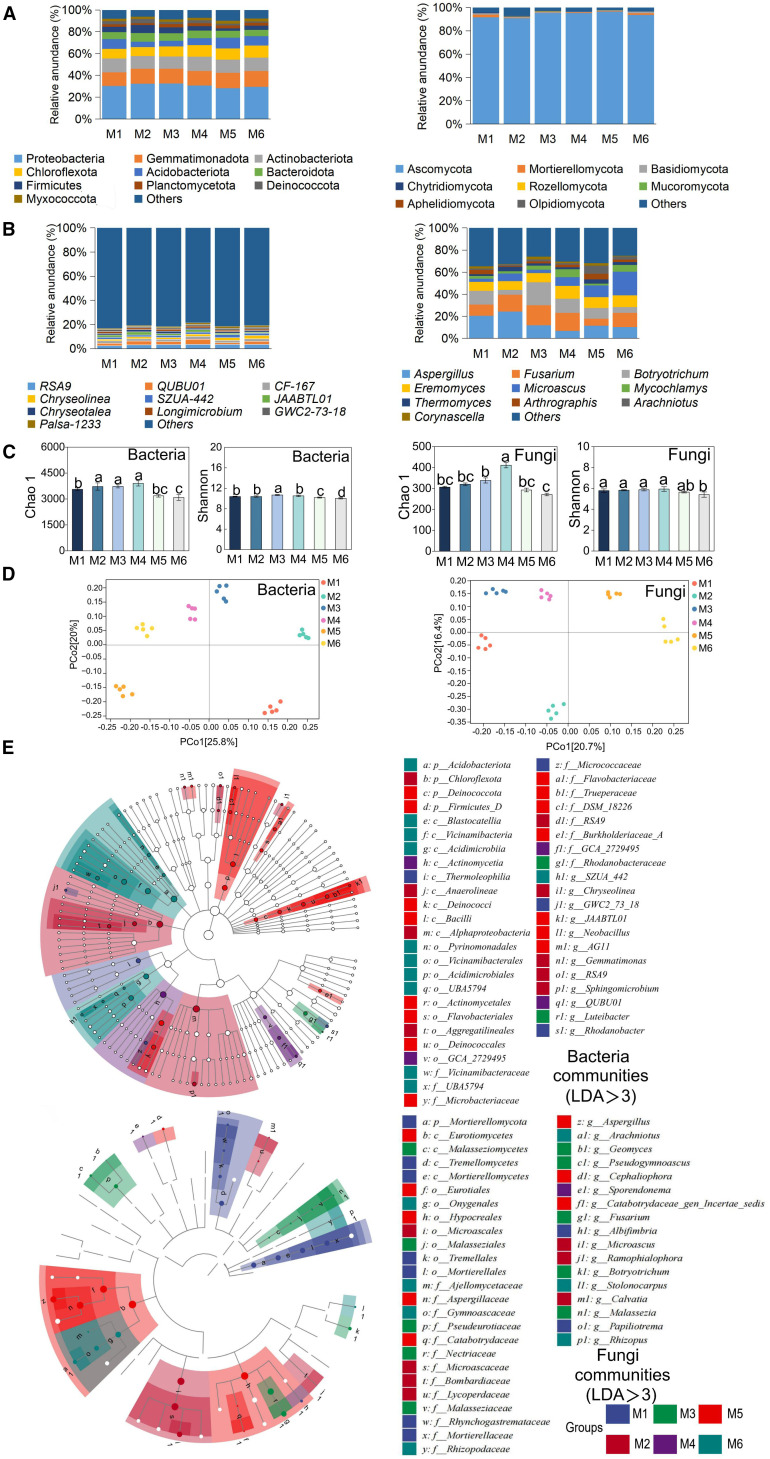
Effects of different treatments on microbial communities. **(A)** Relative abundance of major bacterial (left) and fungal (right) phyla between different treatments. The large circle indicates high abundance. **(B)** Relative abundance of major bacterial (left) and fungal (right) genera between different treatments. **(C)** Comparison of bacterial and fungal community diversity between different treatment areas. **(D)** PCoA analysis of bacterial (left) and fungal (right) communities based on Bray-Curtis distance. **(E)** bacterial and fungal biomarker taxa composition under different treatments. Circles from inside to outside represent taxonomic levels from phylum to genus. Each small circle at a different taxonomic level represents a taxonomic unit at that level, and the size of the circle diameter is proportional to the size of the relative abundance. Species without significant differences are uniformly white. Species names indicated by letters in the figure are shown in the legend on the right, and only species with LDA scores of >3 for differences are shown. Different letters indicate significant differences among treatments (*P* < 0.05, n = 6, Tukey HSD). The treatment codes, M1-M6, are consistent with those in **Figure 1**.

At the phylum level, the dominant bacterial taxa were Proteobacteria, Gemmatimonadota, and Actinobacteria. Proteobacteria had the highest relative abundance (28–32%) and were significantly higher in M3 than in M5 (+14.29%) (*P* < 0.05, n=6; **Figure 3A** and **Supplementary Table S5**). The *QUBU01* was the dominant genus under the M4 treatment and belonged to the Proteobacteria phylum (*P* < 0.05, n=6; **Figure 3B**; **Supplementary Table S7**). The results suggested that treatments M3 and M4 provided more favorable conditions for the growth of Proteobacteria. For fungi, Ascomycota was the predominant phylum and showed higher abundance in M5 (**Figure 3A**; **Supplementary Table S6**).

The Chao1 index of both bacteria and fungi peaked in M4 (26.45% and 51.49% higher than in M6, respectively, *P* < 0.05). The Shannon index of bacteria and fungi was the highest under the M3 and M4 treatments, respectively (*P* < 0.05, n=6; **Figure 3C**). There were significant differences in soil microbial community structure among treatments (**Figure 3D**). Additionally, the alpha diversity of bacteria and fungi (Chao1, Shannon index) exhibited positive correlations with transpiration ratio (Tr), protein, Vitamin C (VC), reducing sugars (Rs), total sugar (Ts), and yield, but a negative correlation with the fruit shape index (FSI) (**Supplementary Figure S4**).

Biomarkers often represent taxa that perform critical ecological functions. The biomarker taxa showed significant differences among the six treatments. For bacteria, the number of biomarker taxa was the highest in M2 (16) and lowest in M3 (2). For fungi, it was the highest in M1 (12) and lowest in M4 (4) (**Figure 3E**). Furthermore, we found that most of these biomarkers belong to the Acidobacteria phylum (bacteria) and the Ascomycota phylum (fungi), indicating that they play a crucial role in the functioning of soil ecosystems.

### 3.4 Molecular ecological network analysis of bacterial and fungal communities

**Figure 4:**
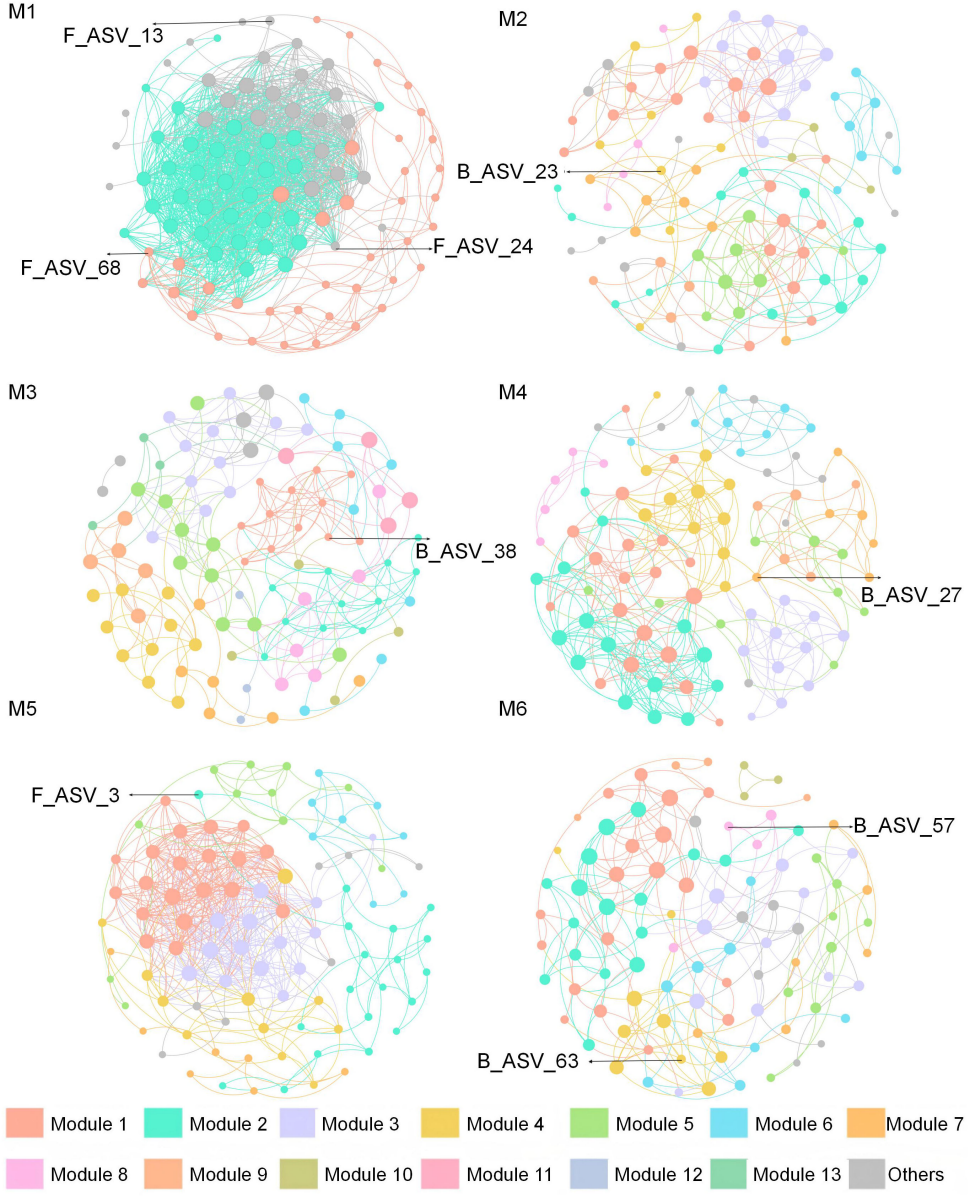
Bacterial and fungal co-occurring networks under each treatment. The treatment codes, M1-M6, are consistent with those in **Figure 1**.

Microbial interactions across the six treatments were analyzed using a molecular ecological network. A total of 2, 10, 13, 9, 7, and 10 major modules were identified within each respective treatment, collectively accounting for over 90% of the network structure (**Figure 4**). Under the M1 treatment, average degree (avgK, 32.55) and average clustering coefficient (avgCC, 0.76) were higher, whereas modularity was significantly lower than in the other treatments. The highest connectedness (Con) index (1.00) occurred under the M4. The positive correlation between fungi and bacteria was generally higher than the negative correlation under all treatments. Its range was distributed between 65.98% (M4) and 94.61% (M5) (**Supplementary Table S9**). Besides, four microbial key taxa were identified at the phylum level, including three bacteria (Firmicutes, Acidobacteriota, and Gemmatimonadota) and one fungus (Ascomycota) (**Supplementary Figure S5**; **Supplementary Table S10**).

### 3.5 Functional gene changes under different treatments

**Figure 5:**
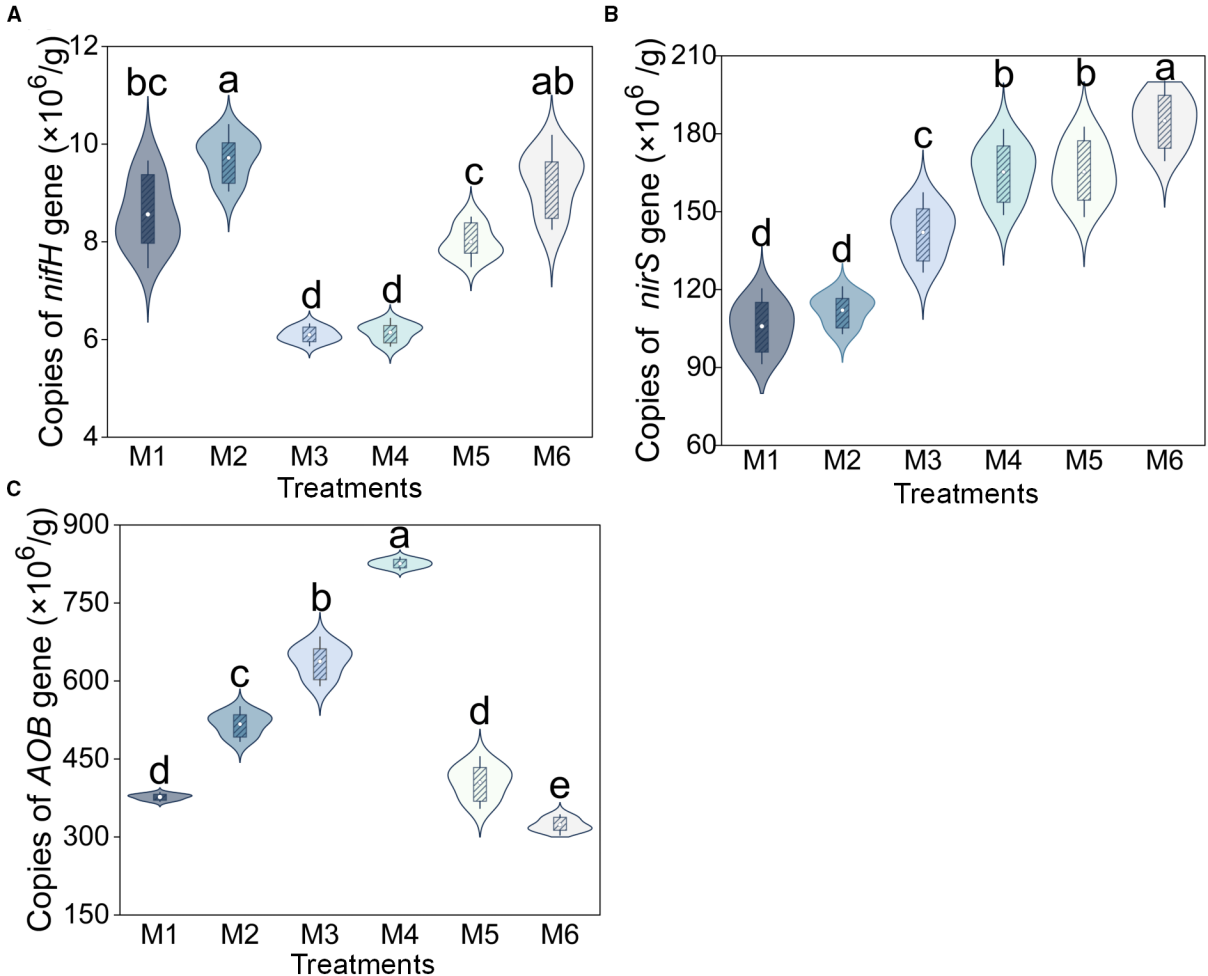
Absolute abundance of **(A)** *nifH*, **(B)** *nirS*, and **(C)** *AOB* gene under different treatments. Different letters indicate significant differences between treatments (*P* < 0.05, n = 6, Tukey HSD). The treatment codes, M1-M6, are consistent with those in **Figure 1**.

The functional genes involved in nitrogen cycling exhibited three distinct trends. The copies of *nifH* showed a bimodal pattern, peaking under M2 treatment with significant increases of 59.46% and 58.10% compared to the lowest levels in M3 and M4 (*P* < 0.05; **Figure 5A**). The copies of *nirS* demonstrated a progressive increase across treatments (*P* < 0.05, n=6; **Figure 5B**). The copy numbers of *AOB* followed a unimodal distribution, reaching their maximum in M4 (825.83 × 10^6^ copies), representing a 155.46% elevation relative to the level in the sludge-free control (M6) (*P* < 0.05, n=6; **Figure 5C**). Furthermore, the average copies of *AOB* genes were orders of magnitude higher than those of *nifH* genes (64.90 times, **Figure 5**).

### 3.6 Driving factors of yield and quality

**Figure 6:**
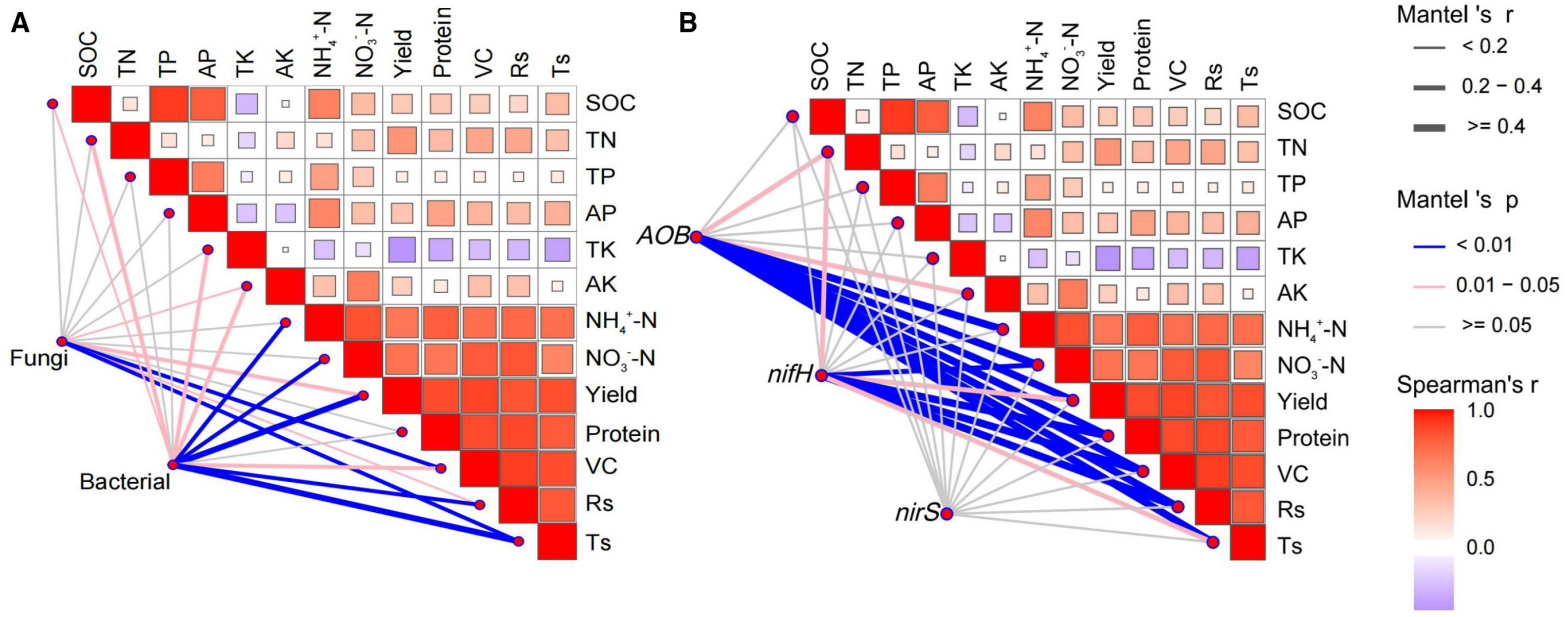
Relationships between **(A)** microbial communities, **(B)** nitrogen cycling genes, and fruit quality, yield, and soil nutrients based on Spearman correlation and Mantel test. SOC, soil organic carbon; TN, total nitrogen; TP, total phosphorus; AP, available phosphorus; TK, total potassium; AK, available potassium; NH_4_^+^-N, ammonium nitrogen; NO_3_^–^N, nitrate nitrogen; VC, Vitamin C, Rs, reducing sugar; Ts, total sugar.

Bacterial and fungal species abundances were strongly correlated with grape performance and soil nutrients. Notably, bacterial species abundances exhibited significant correlations with over half of the indicators (SOC, TN, TK, AK, NH_4_^+^-N, NO_3_^–^N, yield, VC: Vitamin C, Rs: reducing sugars, and Ts: total sugar), demonstrating a particularly strong association with yield (r ≥ 0.4, *P* < 0.01, **Figure 6A**). However, fungi showed significant correlations with only a few indicators (AK, yield, VC, Rs, and Ts). This indicated that bacteria had a greater impact on grape performance and soil nutrients than fungi.

The nitrogen cycle gene *AOB* copies strongly correlated with NH_4_^+^-N, NO_3_^–^N, yield, protein, VC, Rs, and Ts (r ≥ 0.4, *P* < 0.01) and was closely related to TN and AK (0.2 < r < 0.4, *P* < 0.05). Similarly, the copies of *nifH* showed strong correlations with NO_3_^–^N, yield, protein, VC, and Rs (r ≥ 0.4, *P* < 0.01) and were closely related to TN and Ts (0.2 < r < 0.4, *P* < 0.05, **Figure 6B**). In contrast, *nirS* showed no significant correlation with any indicator. In summary, these strong correlations demonstrate the key role of *AOB* gene copies in regulating grape yield and quality (protein, VC, Rs, Ts).

Additionally, soil nutrients (except TK) were positively correlated with grape performance, especially TN, NH_4_^+^-N, and NO_3_^–^N. In conclusion, the results indicated that applying SAH and urea mainly affected grape fruit quality and yield by regulating the bacterial community, *AOB*, and *nifH* gene copies (**Figure 6**).

## 4 Discussion

### 4.1 Effect of SAH-urea co-application on grape performance

Crop stem thickness and leaf area (LA) are associated with high crop yield and quality, and they are key indicators for assessing crop growth and predicting yield (Pei et al., 2024). Plants with thicker stems can store more water and nutrients, and they have high stress resistance and load-bearing capacity (Li et al., 2022b). The LA can regulate photosynthetic efficiency, further affecting yield (Ren et al., 2025). The fruit shape index (FSI), defined as the ratio of longitudinal to transverse diameter, serves as a key morphological indicator in fruit characterization (Croce et al., 2024). The FSI closer to 1 indicates a more orbicular fruit morphology. The contents of sugar, vitamins, and other nutrients in the fruit have a considerable influence on the taste and flavor of the fruit, and protein, Vitamin C (VC), reducing sugar (Rs), and total sugar (Ts), which are generally the main evaluation indices (Zhang et al., 2024b; Balık et al., 2023).

Studies have shown that fertilizer application significantly increased the LA of plants and improved the effective area for photosynthesis, thus affecting yield (Cheng et al., 2025). Homsa et al. (2023) examined garlic growth and found that strategic fertilization produced garlic with a larger LA, more photosynthesis, and the highest tuber growth. Wu et al. (2023b) demonstrated that the use of plant growth regulators altered the FSI of grapes (0.92). The content of VC and soluble sugars in radishes increased by 10.62% and 2.15% after the application of organic and inorganic fertilizers, respectively (Jin et al., 2024). The above results demonstrate a strong link between fertilizer application and crop nutrient content (Hu et al., 2024a).

In our study, applying 60–80% of SAH (M3, M4) achieved the best results for grapevine stem thickness, LA, photosynthesis, FSI, quality, and yield (**Figure 1**; **Supplementary Figure S1**, **Table 1**). This suggests that this dosage range can significantly enhance fruit development and canopy photosynthesis, leading to higher yields and overall plant performance. Moreover, the humic acid and amino acids in SAH can enhance the activity of enzymes involved in sugar metabolism, promote the accumulation and transformation of sugars, and increase the Rs and Ts content in fruits (Yuan et al., 2024). While the efficacy of chemical fertilizers in promoting plant growth is well-established, their overuse has led to a range of environmental issues (Pahalvi et al., 2021). Therefore, developing novel, environmentally friendly fertilizers from waste materials, which can replace or reduce chemical fertilizer application, is crucial for advancing sustainable viticulture. These findings provide strong empirical evidence for significantly reducing reliance on chemical fertilizers while enabling the valuable reuse of waste. To thoroughly evaluate the treatment’s effectiveness, we will next investigate how SAH affects the expression of genes related to fruit shape and quality using molecular approaches.

### 4.2 Effect of SAH-urea co-application on soil nutrient content

Soil nutrients have a strong effect on land productivity, and fertilization can replenish the nutrient stocks in the soil and affect soil fertility and plant growth (Tian et al., 2022). In our study, the contents of TN, NH_4_^+^-N, and NO_3_^–^N were the highest under the M4 treatment (**Supplementary Table S4**). Du et al. (2024) observed that the use of either nitrogen-only fertilizer or nitrogen–phosphorus–potassium compound fertilizer led to significant alterations in soil physicochemical characteristics. The N content was enhanced by 25.41% after N fertilizer application compared with the no fertilizer application. This is in line with our findings (**Supplementary Table S4**; **Figure 2**).

The AP and AK can be directly absorbed by the crop, affect the nutritional status of the crop, and are indicators of the intensity of the soil’s ability to supply P and K (Ye et al., 2024). Extended organic fertilization reportedly promotes P enrichment in soils (Wang et al., 2024a). Jiang et al. (2024) reported that fertilization significantly elevated soil nutrient levels, with AP increasing by 17.12% (nitrogen fertilizers) to 474.74% (nitrogen-phosphorus-organic fertilizers) and AK by 2.90% (nitrogen-phosphorus fertilizers) to 64.40% (nitrogen-phosphorus-organic fertilizers). Notably, the significant increases in AP and AK contents under the M3 and M4 treatments observed in this study did not originate from conventional chemical fertilizers, but rather from the mineralization of organic matter in SAH and its stimulatory effect on soil microbial activity (**Supplementary Table S4**). This organic waste-driven approach to enhancing soil fertility not only offers more sustained benefits but also effectively improves soil structure and mitigates compaction issues associated with sole chemical fertilizer application, representing a more sustainable strategy for nutrient management (Hu et al., 2024b; N’Dri et al., 2023).

### 4.3 Effect of SAH-urea co-application on microbial community

Fertilization can increase soil nutrients, regulate microbial community structure, and improve farm productivity (Fan et al., 2021; Hartmann and Six, 2023). Changes in fertilizer ratios influenced community structure and the distribution of microbial taxa. For example, the dominant bacterial phylum under the M3 treatment was Proteobacteria. Meanwhile, the dominant fungal phylum under M5 treatment was Ascomycota (**Figure 3A**). In addition, the highest microbial diversity indices were observed under the M3 and M4 treatments (**Figure 3C**), which was attributed to soil structure and nutrient conditions regulating microenvironmental heterogeneity, thus directly or indirectly controlling microbial composition (Hu et al., 2025; Laurent et al., 2023).

The biomarker and key taxa of microbiological under different treatments were characterized further by LEfSe analysis and molecular ecological network. There were differences in the biomarker and key taxa of microbiological under different treatments (**Figures 3E**, **4**). Such variation likely arises from differential microbial colonization strategies across distinct soil environments (Zhang et al., 2022). Interestingly, we found that the Acidobacteriota and Ascomycetes belong to both biomarker and key taxa (**Supplementary Figure S5**; **Supplementary Table S10**). The phenomenon is primarily attributed to their unique metabolic complementarity; metabolites (e.g., organic acids) secreted by Ascomycetes facilitate Acidobacteriota proliferation, thereby enhancing soil nitrogen fixation (Kost et al., 2023). Such cross-kingdom interaction markedly improves community stability and nutrient-use efficiency, rendering such taxa ecologically indispensable within microbial networks (Jia et al., 2025). However, considering the dynamic nature of microbial communities, future studies should incorporate microbial data from different application durations to better assess the long-term impacts of SAH on soil microbiota.

Positive correlations in microbial networks can generally be interpreted as interactive symbiotic and mutualistic relationships between species (Liu et al., 2024). Notably, under M5 treatment, microbial cooperation was highest (**Supplementary Table S9**); however, the yield was lower (**Table 1**). Previous studies indicate that microbial cooperation is influenced by environmental nutrient conditions: under low-nutrient conditions, microorganisms enhance cooperation to resist stress and maintain survival (Dai et al., 2022). Thus, the high microbial cooperation observed in the M5 treatment represents a “defensive” strategy prioritizing survival under adverse conditions, rather than directly serving grape growth. This explains the decoupling between cooperation and yield. The M1 treatment exhibited maximal values for both average degree (avgK) and average clustering coefficient (avgCC). This indicates that the microbial network under this treatment has better adaptability and flexibility and is more closely connected in function (Du et al., 2025). It can cope with environmental changes through internal connections, reconnect, and adjust to maintain its functionality and structure with higher robustness and a more stable network (Zhang et al., 2024c; Al Musawi et al., 2023; Puente-Sánchez et al., 2024).

Under M4 treatment, *AOB* exhibited the highest gene copy number (**Figure 5C**), which may be related to SOC and TN content (Zhou et al., 2014; Li et al., 2023). Previous studies indicate that *AOB* abundance correlates positively with soil C, N, and NH_4_^+^-N contents. The elevated SOC and TN levels provide additional growth substrates for *AOB*, thereby enhancing their proliferation (Thion et al., 2016; Li et al., 2017; Song and Niu, 2022). Additionally, it is noteworthy that although the *nifH* gene copies peaked in M2, this trend contrasts sharply with the superior agronomic performance observed in M4. This is because the *nifH* gene copy number is relatively low (**Figure 5**), limiting its potential impact in soil (Li et al., 2022). Moreover, the association between *AOB* gene abundance and agronomic performance was stronger than that of *nifH* (**Figure 6**). The above results collectively indicate that the *nifH* gene is not a key factor influencing grape agronomic performance, while *AOB*-driven nitrification may be a more direct and efficient nitrogen supply pathway in soil, thereby more critically determining the final expression of agronomic performance (Li et al., 2018; Ma et al., 2021a).

### 4.4 Bacterial communities, *AOB*, and *nifH* genes mainly regulate the yield and nutritional quality

Grape yields and their quality are often closely related to microbial species and gene copy number (Guo et al., 2021; Asad et al., 2022). Close associations between bacterial communities, *AOB*, *nifH* genes, and yield and fruit nutritional quality were found following Mantel test analysis (**Figure 6**). Soil bacteria play an important role in breaking down crop residues, mineralizing, and fixing SOC (Shamshitov et al., 2024). Some bacterial populations can inhibit soil pathogen growth, protecting the health of the crop and improving yields (Liang et al., 2025). Wang et al. (2024b) showed that stover returns increased maize yield in arid and semi-arid regions mainly by regulating soil bacterial community abundance. Bacteria are 1.4–5 times more efficient than fungi in using simple organic compounds (Dini et al., 2025). Structural features of bacterial cell membranes enable rapid binding and utilization of small organic molecules in SAH, facilitating metabolic adaptation to maximize nutrient use and thereby influence crop yield (Akhtar et al., 2024). Due to the roles of *AOB* and *nifH* genes in nitrogen cycling, an increase in their gene abundance may imply more efficient nitrogen transformation, positively affecting crop nitrogen use and yield (Ma et al., 2021b). This suggests that changes in bacterial abundance and the number of *AOB* and *nifH* genes stimulated grape development and increased nutrient acquisition, leading to significant increases in key fruit quality indicators (e.g., Ts and Rs) and yield.

The results demonstrate that the combined application of SAH and urea establishes a more efficient and synergistically integrated soil nutrient delivery system. This improvement can be attributed to the optimized resource input strategy achieved through partial substitution of urea with SAH derived from organic waste. Therefore, this study not only presents an effective fertilization strategy but also highlights the significant potential of organic wastes in reducing chemical fertilizer application and promoting nutrient recycling, thereby providing important theoretical and practical support for sustainable viticulture.

## 5 Conclusions

**Figure 7:**
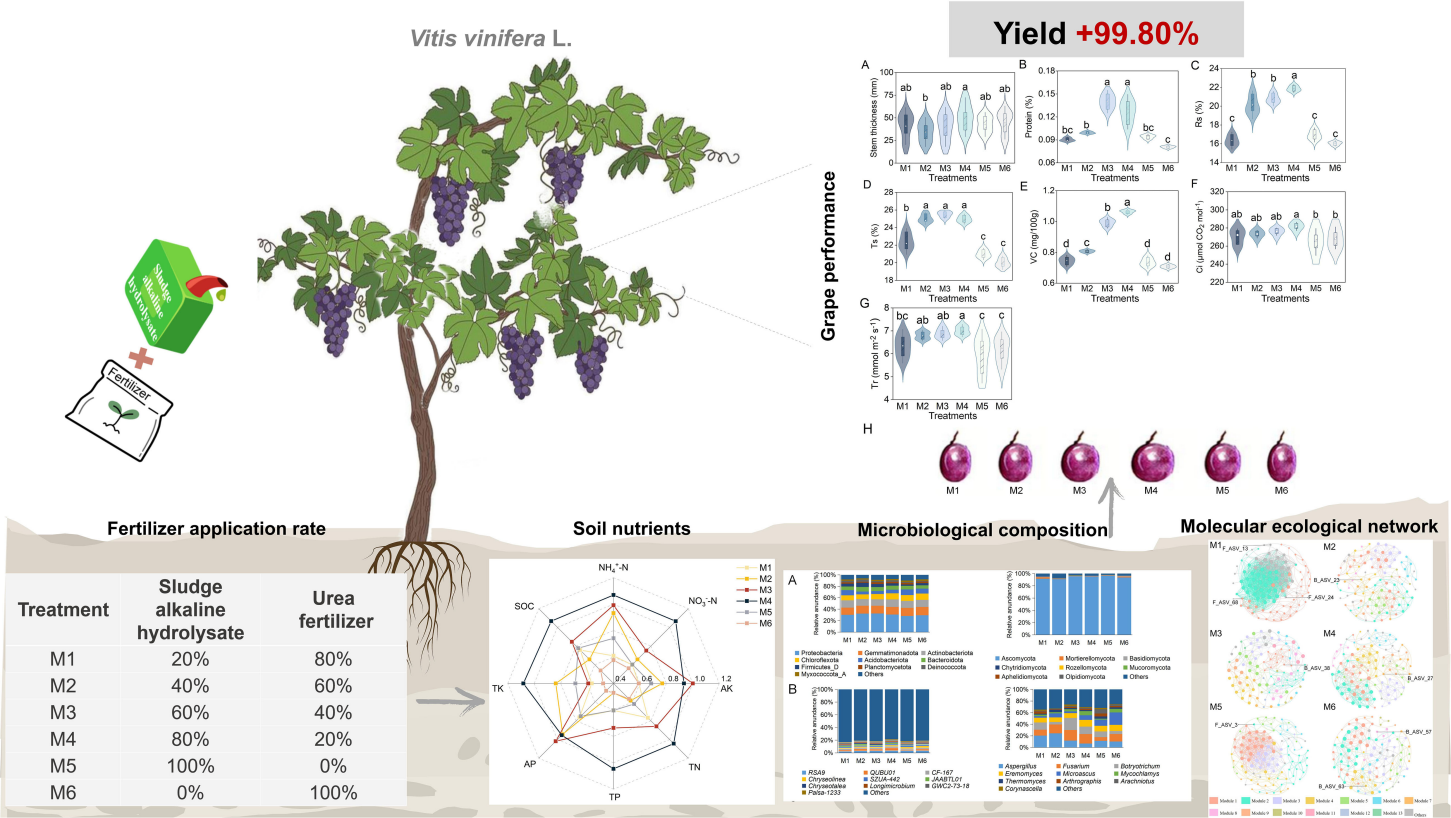
A concept figure illuminated the effects of varying proportions of SHA on grape performance, soil nutrients, and microbial communities. The application of SHA enhances soil nutrient availability, modulates microbial community composition, and accelerates plant growth and nutrient accumulation, thereby increasing yield. The treatment codes, M1-M6, are consistent with those in **Figure 1**.

Our study systeatically evaluated the influence of varying proportions of SAH and urea on multiple aspects of grape production systems (**Figure 7**). At SAH-to-urea fertilizer ratios of 60:40 and 80:20, grape performance, soil nutrients, microbial community structure, and nitrogen cycle functional genes reached optimal levels. The molecular ecological network showed that Firmicutes, Acidobacteriota, Gemmatimonadota, and Ascomycota were key taxa. Notably, bacteria and fungi showed strong cooperation (over 50%), which significantly enhanced the microbial network’s adaptability, modularity, and species coexistence. Mantel test showed that bacterial community and *AOB* and *nifH* gene copies are key factors regulating grape quality and yield. These findings demonstrate that SAH functions as an ecological regulator in grape cultivation, providing new insights for sustainable agricultural development.

## Data Availability

The original contributions presented in the study are included in the article/**Supplementary Material**. Further inquiries can be directed to the corresponding author.
