# Supplementary material for: Blending sludge alkaline hydrolysate and urea affects grape yield and quality by regulating soil bacterial communities
Source: Front Plant Sci. 2025 Sep 30;16:1665661. doi: 10.3389/fpls.2025.1665661 (PMC12518296; doi:10.3389/fpls.2025.1665661)
Supplement: Supplementary file 1 [file DataSheet1.docx]

**Blending sludge alkaline hydrolysate and urea affects grape yield and quality by regulating soil bacterial communities**

Donghe Xue^a,b^, Yan yang^a^, Huofeng Zhang^a^, Yijie Quan^a^, Zejin Li^c^, Zixu Li^a^, Wei Wang^a^, Huijuan Bo^a^*, Dongsheng Jin^a,b^*, Minggang Xu^a,b^, Qiang Zhang^a,b^, Zhiping Yang^a,b^

^a^ *College of Resources and Environment, Shanxi Agricultural University, Taiyuan 030031, China*

^b^ *Soil Health Laboratory in Shanxi Province, Taiyuan 030031, China*^c^ *College of Forestry, Beijing Forestry University, Beijing 100083, China*

* Corresponding author. Huijuan Bo

Tel/Fax: +86-0351-7639388

E-mail address: [bohuijuan@sxau.edu.cn](mailto:sxdxjds@126.com)

Dongsheng Jin

E-mail address: sxdxjds@126.com


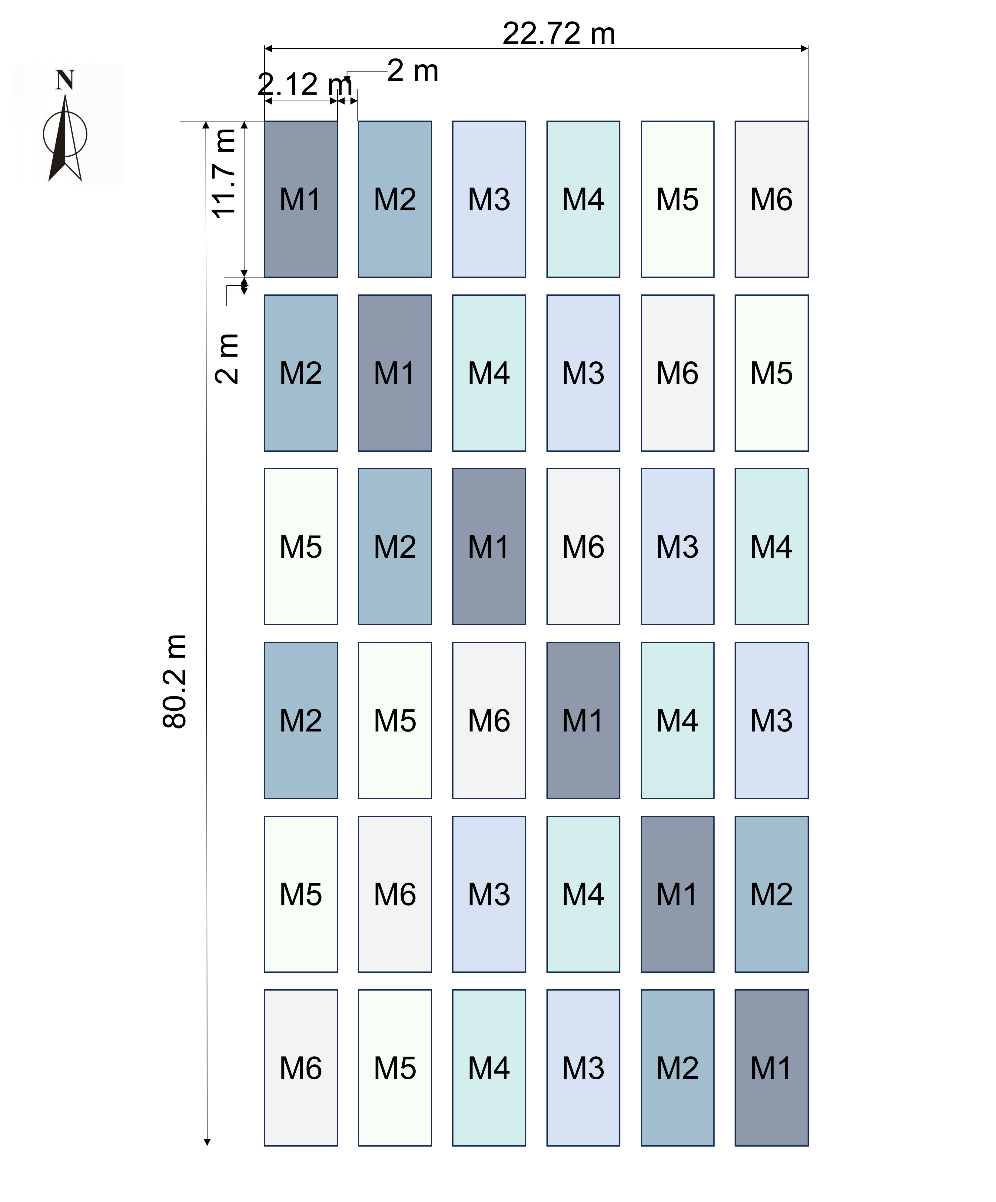


**FIGURE S1**

Test plot size and distribution map. The treatments included 20% SAH + 80% urea (M1), 40% SAH + 60% urea (M2), 60% SAH + 40% urea (M3), 80% SAH + 20% urea (M4), pure SAH (M5), and pure urea (M6). SAH—sludge alkaline hydrolysate. Each plot has 10 grapevines, planted at a spacing of 1.30 × 2.12 m.

**
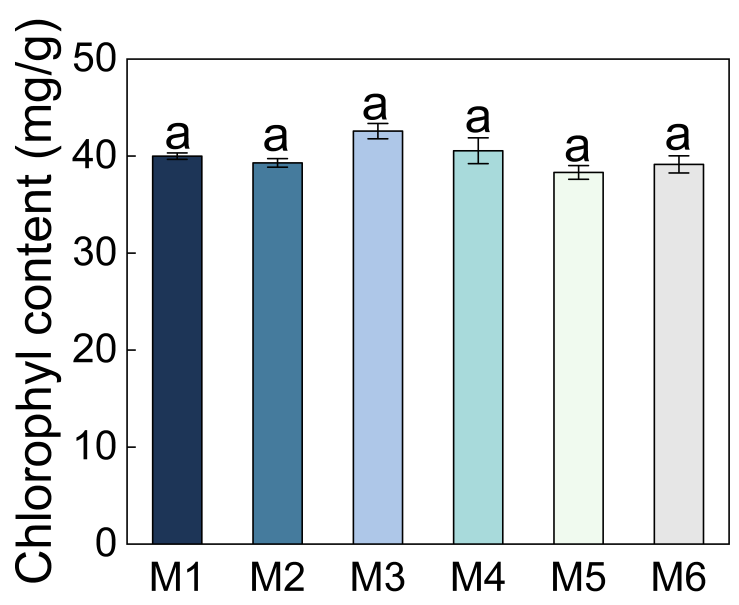
**

**FIGURE S2**

Chlorophyll content under different treatments. Different letters indicate significant differences among treatments (*P* < 0.05, n = 6, Tukey HSD). The treatments included 20% SAH + 80% urea (M1), 40% SAH + 60% urea (M2), 60% SAH + 40% urea (M3), 80% SAH + 20% urea (M4), pure SAH (M5), and pure urea (M6). SAH—sludge alkaline hydrolysate.


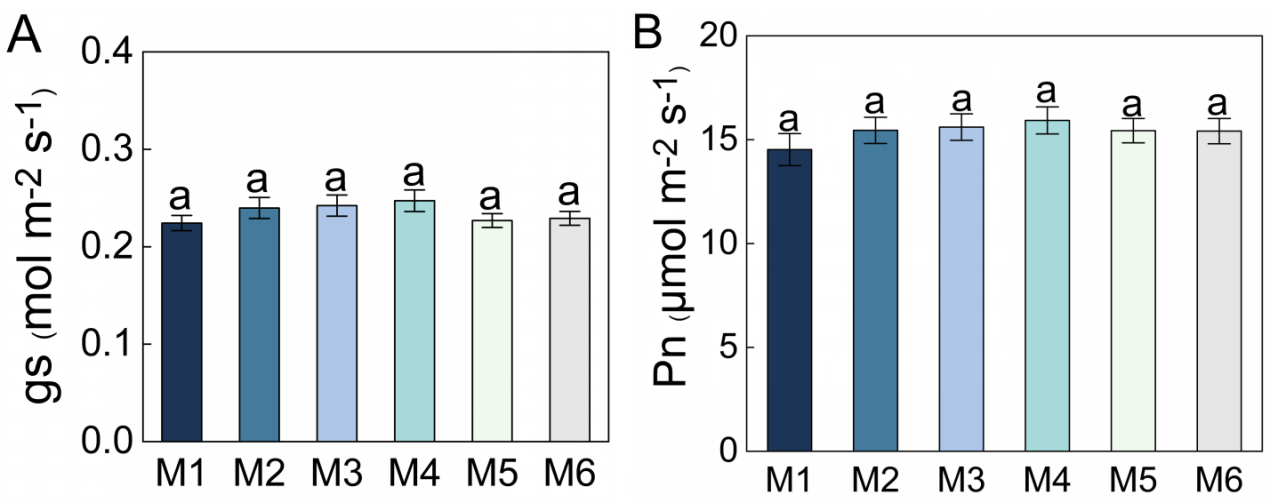


**FIGURE S3**

Photosynthetic characteristics under different treatments. (A) gs—stomatal conductance, (B) Pn—net photosynthetic rate. Different letters indicate significant differences among treatments (*P* < 0.05, n = 6, Tukey HSD). The treatments included 20% SAH + 80% urea (M1), 40% SAH + 60% urea (M2), 60% SAH + 40% urea (M3), 80% SAH + 20% urea (M4), pure SAH (M5), and pure urea (M6). SAH—sludge alkaline hydrolysate.

**
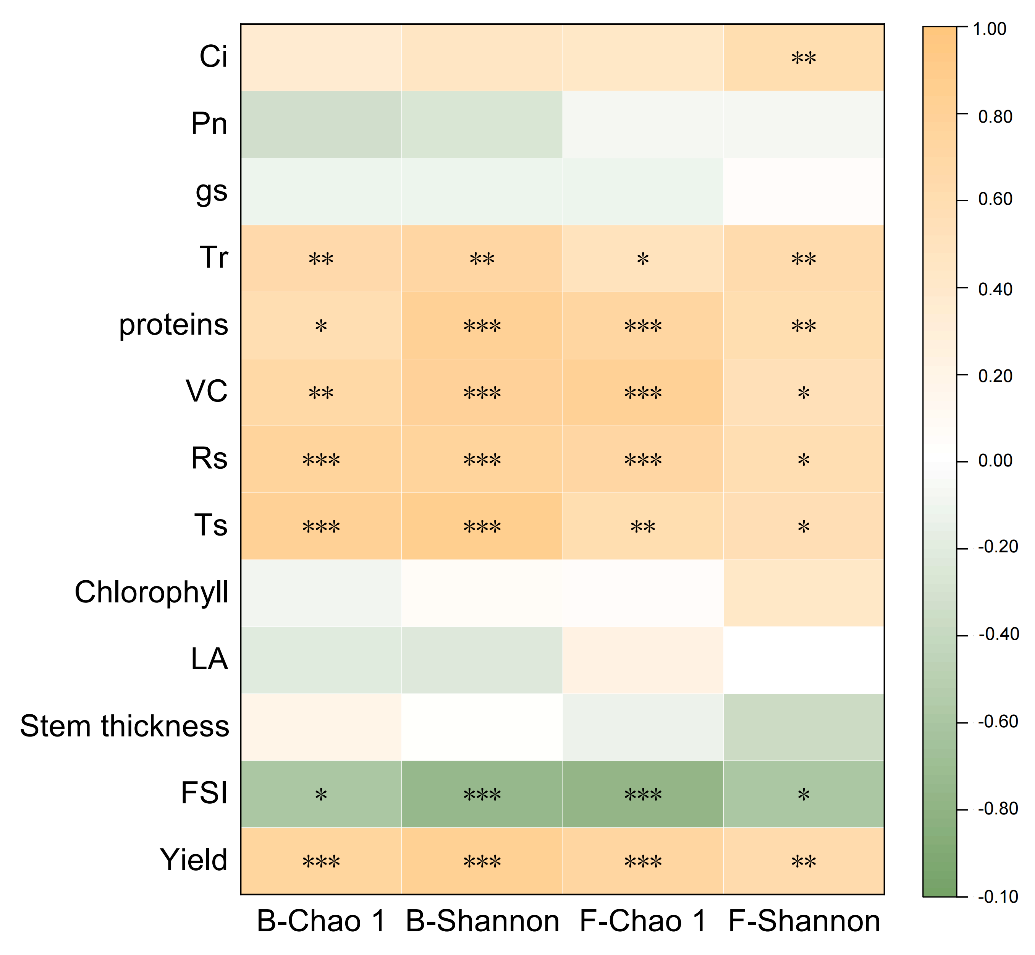
**

**FIGURE S4**

The correlation analysis between microbial diversity indices and grape performance. Ci—intercellular CO_2_ concentration, Pn—net photosynthetic rate, gs—stomatal conductance, Tr—transpiration ratio, VC—Vitamin. C, Rs—reducing sugar, Ts—total sugar, LA—leaf area, FSI—fruit shape index, Y—yield, B—bacterial, F—fungal. *: *P* ≤ 0.01, **: *P* ≤ 0.05, ***: *P* ≤ 0.001.


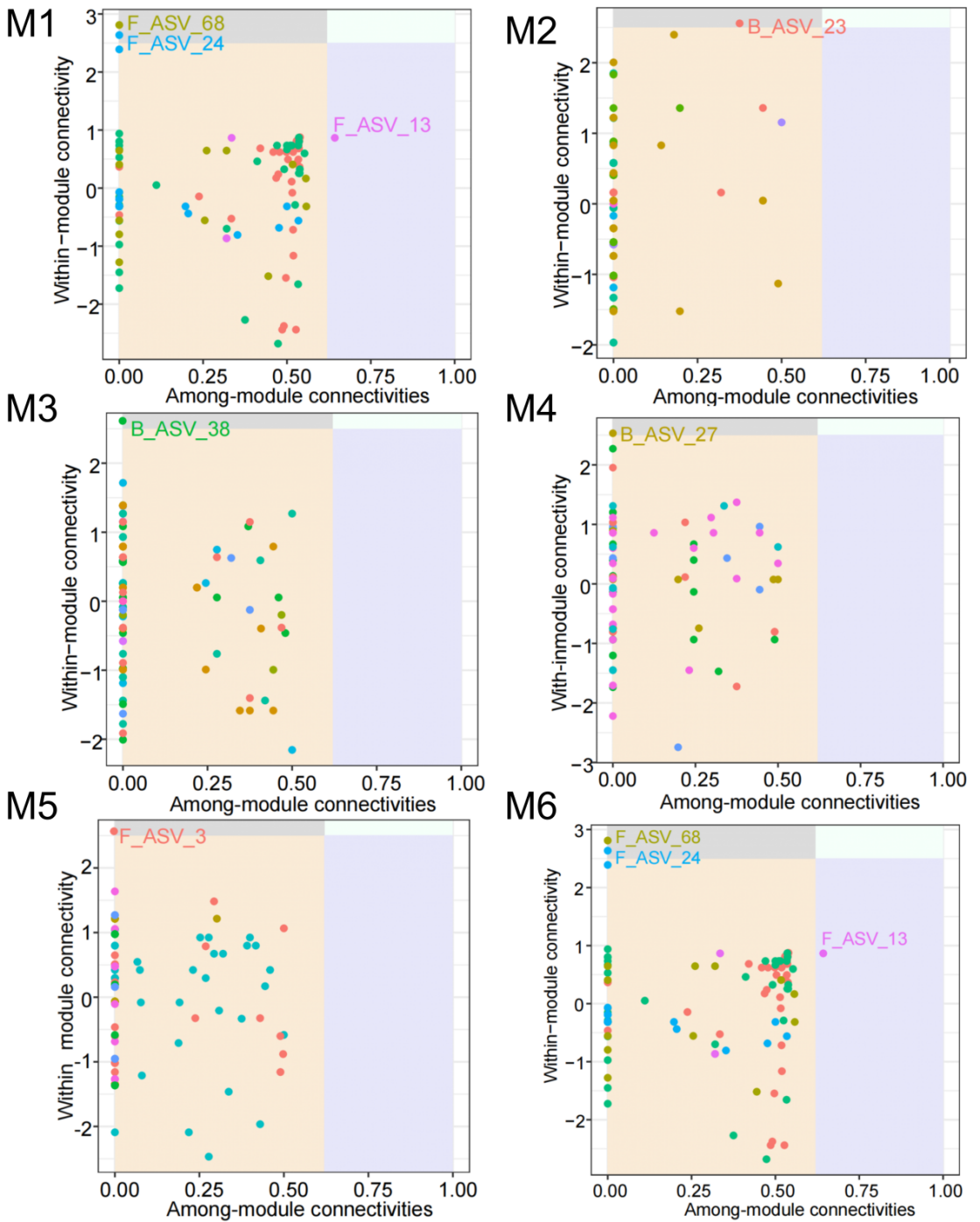


**FIGURE S5**

Microbial key taxa under different fertilization treatments. The treatments included 20% SAH + 80% urea (M1), 40% SAH + 60% urea (M2), 60% SAH + 40% urea (M3), 80% SAH + 20% urea (M4), pure SAH (M5), and pure urea (M6). SAH—sludge alkaline hydrolysate.

**TABLE S1** Composition of Sludge Alkaline Hydrolysate

| Parameter | Content | Parameter | Content |
| --- | --- | --- | --- |
| SOC (g/L) | 153.49 ± 9.37 | N (g/L) | 44.08 ± 0.32 |
| HA (g/L) | 8.11 ± 0.08 | P (g/L) | 316.00 ± 18.90 |
| Protein (g/L) | 63.60 ± 1.90 | K (g/L) | 8.75 ± 0.05 |
| Peptides (g/L) | 116.70 ± 12.10 | Ca (g/L) | 55.30 ± 3.70 |
| FAA (g/L) | 54.67 ± 5.21 | Pb (mg/L) | 3.00 ± 0.10 |
| NH_4_^+^-N (g/L) | 1.28 ± 0.13 | Hg (μg/L) | 66.20 ± 5.90 |
| NO_3_^-^-N (g/L) | 0.19 ± 0.04 | Cd (μg/L) | 12.30 ± 3.80 |

Note: SOC—soil organic carbon; HA—humic acid; FAA—free amino acids.

**TABLE S2** Doses of fertilizers and nutrients for the different treatments (kg*/*ha)

| Treatment | Nitrogen application rate | | Fertilizer application amount | |
| --- | --- | --- | --- | --- |
|  | Nitrogen fertilizer  (kg/ha) | Concentrated solution  (L/ha) | Nitrogen fertilizer  (kg/ha) | Concentrated solution  (L/ha) |
| M1 | 141.48 | 35.37 | 304.92 | 384.77 |
| M2 | 106.11 | 70.74 | 228.69 | 769.52 |
| M3 | 70.74 | 106.11 | 152.46 | 1154.28 |
| M4 | 35.37 | 141.48 | 76.23 | 1539.03 |
| M5 | 0.00 | 176.85 | 0.00 | 1923.80 |
| M6 | 176.85 | 0.00 | 381.14 | 0.00 |

Note： The treatments included 20% SAH + 80% urea (M1), 40% SAH + 60% urea (M2), 60% SAH + 40% urea (M3), 80% SAH + 20% urea (M4), pure SAH (M5), and pure urea (M6). SAH—sludge alkaline hydrolysate. The total nitrogen application rate is 176.85 kg/ha. The nitrogen content of nitrogen fertilization is 46.40%, and the SAH nitrogen content of sludge is 7.33%.

**TABLE S3** Primer information used in the experiment

| ID | Primer name | Sequence (5'- 3') |
| --- | --- | --- |
| *AOB* | amoA-1F | GGGGTTTCTACTGGTGGT |
|  | amoA-2R | CCCCTCKGSAAAGCCTTCTTC |
| *nirS* | cd3aF | GTSAACGTSAAGGARACSGG |
|  | R3cdR | GASTTCGGRTGSGTCTTGA |
| *nifH* | PolyF | TGCGAYCCSAARGCBGACTC |
|  | PolyR | ATSGCCATCATYTCRCCGGA |

**TABLE S4** Soil nutrient content under different treatments

| Treatment | SOC | TN | TP | AP | TK | AK | NH_4_^+^-N | NO_3_^–^-N |
| --- | --- | --- | --- | --- | --- | --- | --- | --- |
|  | (g/kg) | (g/kg) | (g/kg) | (mg/kg) | (g/kg) | (mg/kg) | (mg/kg) | (mg/kg) |
| M1 | 11.02 ± 0.79ab | 1.14 ± 0.14abc | 1.24 ±0.09a | 60.54 ± 4.35ab | 20.28 ± 1.16b | 428.14±30.79c | 3.84 ± 0.10b | 32.45 ± 0.87cd |
| M2 | 10.57 ± 0.95b | 1.04 ± 0.09bc | 1.21 ± 0.10a | 65.40 ± 3.62a | 21.51 ± 0.58ab | 556.31 ± 47.16b | 5.30 ± 0.38a | 37.69 ± 4.32c |
| M3 | 11.42 ± 1.31ab | 1.19 ± 0.10ab | 1.27 ± 0.07a | 67.43 ± 7.73a | 20.40 ± 1.12b | 723.55 ± 65.12a | 5.57 ± 0.64a | 46.22 ± 3.92b |
| M4 | 12.42 ± 1.05a | 1.29 ± 0.04a | 1.34 ± 0.12a | 65.68 ± 1.91a | 22.62 ± 1.25a | 675.20 ± 18.23a | 5.92 ± 0.50a | 74.67 ± 5.37a |
| M5 | 11.11 ± 0.62ab | 1.06 ± 0.06bc | 1.24 ± 10.14a | 60.16 ± 5.41ab | 20.84 ± 0.66ab | 495.66 ± 56.83ab | 4.44 ± 0.25b | 32.77 ± 1.81cd |
| M6 | 10.03 ± 0.27b | 1.03 ± 0.02c | 1.21 ± 0.03a | 52.78 ± 1.42b | 19.89 ± 1.39b | 427.71 ± 23.64c | 3.69 ± 0.34b | 29.10 ± 2.62d |

Note: SOC—soil organic carbon; TN—total nitrogen; TP—total phosphorus; AP—available phosphorus; TK—total potassium; AK—available potassium; NH_4_^+^-N—ammonium nitrogen; NO_3_^–^-N—nitrate nitrogen. Different letters indicate significant differences among treatments (*P* < 0.05, n = 6, Tukey HSD). The treatments included 20% SAH + 80% urea (M1), 40% SAH + 60% urea (M2), 60% SAH + 40% urea (M3), 80% SAH + 20% urea (M4), pure SAH (M5), and pure urea (M6). SAH—sludge alkaline hydrolysate.

**TABLE S5** Top ten species in relative abundance at the bacterial phylum level under different treatments

| Treatment | M1 | M2 | M3 | M4 | M5 | M6 |
| --- | --- | --- | --- | --- | --- | --- |
| Proteobacteria | 0.30 ± 0.01ab | 0.32 ± 0.03a | 0.32 ± 0.02a | 0.31 ± 0.01ab | 0.28 ± 0.01b | 0.30 ± 0.00ab |
| Gemmatimonadota | 0.13 ± 0.01b | 0.14 ± 0.01ab | 0.14 ± 0.01ab | 0.13 ± 0.01ab | 0.14 ± 0.00ab | 0.15 ± 0.01a |
| Actinobacteriota | 0.13 ± 0.01ab | 0.12 ± 0.00bc | 0.11 ± 0.01c | 0.13 ± 0.01a | 0.12 ± 0.01abc | 0.12 ± 0.01abc |
| Chloroflexota | 0.09 ± 0.01c | 0.08 ± 0.01c | 0.09 ± 0.01bc | 0.11 ± 0.00a | 0.10 ± 0.01ab | 0.11 ± 0.00a |
| Acidobacteriota | 0.09 ± 0.01b | 0.05 ± 0.00d | 0.05 ± 0.00d | 0.06 ± 0.01c | 0.10 ± 0.00a | 0.09 ± 0.00b |
| Bacteroidota | 0.06 ± 0.01b | 0.08 ± 0.00a | 0.07 ± 0.02ab | 0.07 ± 0.00ab | 0.06 ± 0.00b | 0.06 ± 0.00b |
| Firmicutes | 0.05 ± 0.00b | 0.07 ± 0.00a | 0.05 ± 0.00b | 0.04 ± 0.00c | 0.02 ± 0.00d | 0.04 ± 0.00c |
| Planctomycetota | 0.02 ± 0.00b | 0.01 ± 0.00c | 0.03 ± 0.00a | 0.03 ± 0.00ab | 0.03 ± 0.00ab | 0.03 ± 0.00ab |
| Deinococcota | 0.03 ± 0.00b | 0.04 ± 0.01a | 0.02 ± 0.00cd | 0.03 ± 0.00bc | 0.01 ± 0.00de | 0.01 ± 0.00e |
| Myxococcota | 0.02 ± 0.00bc | 0.02 ± 0.00c | 0.02 ± 0.00bc | 0.02 ± 0.00bc | 0.03 ± 0.00a | 0.03 ± 0.00ab |

Note: Different letters indicate significant differences among treatments (*P* < 0.05, n = 6, Tukey HSD). The treatments included 20% SAH + 80% urea (M1), 40% SAH + 60% urea (M2), 60% SAH + 40% urea (M3), 80% SAH + 20% urea (M4), pure SAH (M5), and pure urea (M6). SAH—sludge alkaline hydrolysate.

| **TABLE S6** Top eight species in relative abundance at the fungal phylum level under different treatments | | | | | | |
| --- | --- | --- | --- | --- | --- | --- |
|  | M1 | M2 | M3 | M4 | M5 | M6 |
| Ascomycota | 0.92 ± 0.02a | 0.91 ± 0.06a | 0.95 ± 0.02a | 0.95 ± 0.02a | 0.96 ± 0.01a | 0.94 ± 0.03a |
| Mortierellomycota | 0.02 ± 0.01a | 0.01 ± 0.00c | 0.00 ± 0.00c | 0.01 ± 0.00c | 0.01 ± 0.00c | 0.01 ± 0.00b |
| Basidiomycota | 0.01 ± 0.00a | 0.01 ± 0.00ab | 0.01 ± 0.00ab | 0.00 ± 0.00c | 0.00 ± 0.00c | 0.01 ± 0.01a |
| Chytridiomycota | 0.01 ± 0.01a | 0.00 ± 0.01a | 0.00 ± 0.00a | 0.00 ± 0.00a | 0.00 ± 0.00a | 0.00 ± 0.00a |
| Rozellomycota | 0.00 ± 0.00b | 0.00 ± 0.00ab | 0.00 ± 0.00ab | 0.00 ± 0.00b | 0.00 ± 0.00b | 0.00 ± 0.00b |
| Mucoromycota | 0.00 ± 0.00a | 0.00 ± 0.00a | 0.00 ± 0.00a | 0.00 ±0.00a | 0.00 ± 0.00a | 0.00 ± 0.00a |
| Aphelidiomycota | 0.00 ± 0.00a | 0.00 ± 0.00a | 0.00 ± 0.00a | 0.00 ± 0.00a | 0.00 ± 0.00a | 0.00 ± 0.00a |
| Olpidiomycota | 0.00 ± 0.00a | 0.00 ± 0.00a | 0.00 ± 0.00a | 0.00 ± 0.00a | 0.00 ± 0.00a | 0.00 ± 0.00a |

Note: Different letters indicate significant differences among treatments (*P* < 0.05, n = 6, Tukey HSD). The treatments included 20% SAH + 80% urea (M1), 40% SAH + 60% urea (M2), 60% SAH + 40% urea (M3), 80% SAH + 20% urea (M4), pure SAH (M5), and pure urea (M6). SAH—sludge alkaline hydrolysate.

| **TABLE S7** Top ten species in relative abundance at the bacterial genus level under different treatments | | | | | | |
| --- | --- | --- | --- | --- | --- | --- |
|  | M1 | M2 | M3 | M4 | M5 | M6 |
| *RSA9* | 0.02 ± 0.00c | 0.03 ± 0.00b | 0.03 ± 0.00ab | 0.03 ± 0.00ab | 0.03 ± 0.00ab | 0.04 ± 0.00a |
| *QUBU01* | 0.02 ± 0.00d | 0.03 ± 0.00b | 0.03 ± 0.00bc | 0.04 ± 0.00a | 0.02 ± 0.00e | 0.02 ± 0.00cd |
| *CF-167* | 0.02 ± 0.00bc | 0.02 ± 0.00c | 0.02 ± 0.00c | 0.02 ± 0.00a | 0.02 ± 0.00bc | 0.02 ± 0.00ab |
| *Chryseolinea* | 0.01 ± 0.00c | 0.02 ± 0.00cd | 0.02 ± 0.00c | 0.02 ± 0.00c | 0.02 ± 0.00b | 0.03 ± 0.00a |
| *SZUA-442* | 0.02 ± 0.00bc | 0.02 ± 0.00c | 0.02 ± 0.00bc | 0.02 ± 0.00bc | 0.03 ± 0.00a | 0.02 ± 0.00b |
| *JAABTL01* | 0.02 ± 0.00bc | 0.03 ± 0.00a | 0.01 ± 0.00cd | 0.02 ± 0.00b | 0.01 ± 0.00de | 0.01 ± 0.00e |
| *Chryseotalea* | 0.01 ± 0.00c | 0.02 ± 0.00ab | 0.02 ± 0.00a | 0.01 ± 0.00c | 0.01 ± 0.00bc | 0.01 ± 0.00c |
| *Longimicrobium* | 0.01 ± 0.00ab | 0.01 ± 0.00b | 0.02 ± 0.00a | 0.02 ± 0.00a | 0.01 ± 0.00b | 0.01 ± 0.00b |

Note: Different letters indicate significant differences among treatments (*P* < 0.05, n = 6, Tukey HSD). The treatments included 20% SAH + 80% urea (M1), 40% SAH + 60% urea (M2), 60% SAH + 40% urea (M3), 80% SAH + 20% urea (M4), pure SAH (M5), and pure urea (M6). SAH—sludge alkaline hydrolysate.

| **TABLE S8** Top ten species in relative abundance at the fungal genus level under different treatments | | | | | | |
| --- | --- | --- | --- | --- | --- | --- |
|  | M1 | M2 | M3 | M4 | M5 | M6 |
| *Aspergillus* | 0.20 ± 0.02a | 0.24 ± 0.01b | 0.12 ± 0.03c | 0.07 ± 0.02d | 0.11 ± 0.02c | 0.10 ± 0.03cd |
| *Fusarium* | 0.10 ± 0.01c | 0.15 ± 0.03ab | 0.18 ± 0.02a | 0.16 ± 0.03ab | 0.06 ± 0.01 d | 0.13 ± 0.02bc |
| *Botryotrichum* | 0.12 ± 0.01b | 0.04 ± 0.02d | 0.21 ± 0.06a | 0.13 ± 0.00b | 0.10 ± 0.03bc | 0.05 ± 0.01cd |
| *Eremomyces* | 0.08 ± 0.03a | 0.08 ± 0.04a | 0.08 ± 0.01a | 0.12 ± 0.02a | 0.10 ± 0.02a | 0.11 ± 0.02a |
| *Microascus* | 0.03 ± 0.01c | 0.07 ± 0.01b | 0.03 ± 0.01c | 0.08 ± 0.04b | 0.10 ± 0.01b | 0.21 ± 0.02a |
| *Mycochlamys* | 0.02 ± 0.01b | 0.02 ± 0.01b | 0.01 ± 0.02b | 0.07 ± 0.02a | 0.02 ± 0.00b | 0.06 ± 0.01a |
| *Thermomyces* | 0.02 ± 0.00b | 0.04 ± 0.01a | 0.02 ± 0.01b | 0.02 ± 0.01b | 0.04 ± 0.01a | 0.03 ± 0.01ab |
| *Arthrographis* | 0.04 ± 0.01ab | 0.01 ± 0.00c | 0.02 ± 0.01bc | 0.02 ± 0.01bc | 0.05 ± 0.02a | 0.02 ± 0.01bc |
| *Arachniotus* | 0.01 ± 0.00b | 0.01 ± 0.00b | 0.02 ± 0.01b | 0.01 ± 0.00b | 0.07 ± 0.06a | 0.03 ± 0.00b |
| *Corynascella* | 0.02 ± 0.01ab | 0.01 ± 0.01b | 0.03 ± 0.02a | 0.01 ± 0.00ab | 0.02 ± 0.01ab | 0.01 ± 0.00b |

Note: Different letters indicate significant differences among treatments (*P* < 0.05, n = 6, Tukey HSD). The treatments included 20% SAH + 80% urea (M1), 40% SAH + 60% urea (M2), 60% SAH + 40% urea (M3), 80% SAH + 20% urea (M4), pure SAH (M5), and pure urea (M6). SAH—sludge alkaline hydrolysate.

| **TABLE S9** The topological properties of networks in soil under different treatments | | | | | | | | | | | | |
| --- | --- | --- | --- | --- | --- | --- | --- | --- | --- | --- | --- | --- |
| Network Indexes | Empirical Network Indexes | | | | | | Random Network Indexes | | | | | |
|  | M1 | M2 | M3 | M4 | M5 | M6 | M1 | M2 | M3 | M4 | M5 | M6 |
| Total nodes | 99 | 95 | 98 | 98 | 97 | 95 | n.a | n.a | n.a | n.a | n.a | n.a |
| Total links | 1611 | 236 | 266 | 338 | 538 | 213 | n.a | n.a | n.a | n.a | n.a | n.a |
| RMT cutoff | 0.94 | 0.94 | 0.94 | 0.94 | 0.94 | 0.94 | n.a | n.a | n.a | n.a | n.a | n.a |
| Average degree (avgK) | 32.55 | 4.97 | 5.43 | 6.90 | 11.09 | 4.48 | n.a | n.a | n.a | n.a | n.a | n.a |
| Average clustering coefficient (avgCC) | 0.76 | 0.52 | 0.49 | 0.60 | 0.67 | 0.48 | 0.64±0.01 | 0.06±0.01 | 0.06±0.01 | 0.10±0.01 | 0.34±0.02 | 0.05±0.01 |
| Positive correlation ratio | 76.17% | 72.03% | 71.80% | 65.98% | 94.61% | 69.01% | n.a | n.a | n.a | n.a | n.a | n.a |
| Negative correlation ratio | 23.83% | 27.97% | 28.20% | 34.02% | 5.39% | 30.99% | n.a | n.a | n.a | n.a | n.a | n.a |
| Connectedness (Con) | 0.92 | 0.45 | 0.45 | 1.00 | 0.34 | 0.86 | 1.00±0.00 | 0.99±0.02 | 0.99±0.02 | 1.00±0.00 | 1.00±0.00 | 0.99±0.01 |
| Efficiency | 0.65 | 0.90 | 0.90 | 0.94 | 0.68 | 0.96 | 0.68±0.00 | 0.96±0.00 | 0.95±0.00 | 0.94±0.00 | 0.90±0.00 | 0.96±0.00 |
| Modularity(fast_greedy) | 0.15 | 0.69 | 0.72 | 0.64 | 0.26 | 0.71 | 0.07±0.01 | 0.39±0.01 | 0.37±0.01 | 0.31±0.01 | 0.18±0.01 | 0.42±0.01 |
| Note：n.a indicates that there is no data available in the randomized algorithm. | | | | | | | | | | | | |

**TABLE S10** The classification of key taxa identified by the microbial network

| Treatment | Key taxa | Kingdom | Phylum | Genus |
| --- | --- | --- | --- | --- |
| M1 | ASV_13 | Fungi | Ascomycota | *unclassified_Ascomycota* |
|  | ASV_68 | Fungi | Ascomycota | *Talaromyces* |
|  | ASV_24 | Fungi | Ascomycota | *Fusarium* |
| M2 | ASV_3 | Bacteria | Firmicutes | *unclassified_DSM-18226* |
| M3 | ASV_38 | Bacteria | Gemmatimonadota | *Gemmatimonas* |
| M4 | ASV_27 | Bacteria | Gemmatimonadota | *RSA9* |
| M5 | ASV_3 | Fungi | Ascomycota | *Aspergillus* |
| M6 | ASV_63 | Bacteria | Acidobacteriota | *Luteitalea* |
|  | ASV_57 | Bacteria | Gemmatimonadota | *Gemmatimonas* |
